# Comprehensive analysis of *formin* gene family highlights candidate genes related to pollen cytoskeleton and male fertility in wheat (*Triticum aestivum* L.)

**DOI:** 10.1186/s12864-021-07878-7

**Published:** 2021-07-24

**Authors:** Wen-jing Duan, Zi-han Liu, Jian-fang Bai, Shao-hua Yuan, Yan-mei Li, Feng-kun Lu, Tian-bao Zhang, Jia-hui Sun, Feng-ting Zhang, Chang-ping Zhao, Li-ping Zhang

**Affiliations:** 1 Beijing Engineering and Technique Research Center for Hybrid Wheat, Beijing Academy of Agriculture and Forestry Sciences, Beijing, 100097 China; 2 The Municipal Key Laboratory of Molecular Genetic of Hybrid Wheat, Beijing, 100097 China; 3 College of Life Science, Capital Normal University, Beijing, 100048 China

**Keywords:** Wheat, Thermo-sensitive genic male sterile, *Formin* gene family, Cytoskeleton, MiRNA

## Abstract

**Background:**

Formin, a highly conserved multi-domain protein, interacts with microfilaments and microtubules. Although specifically expressed *formin* genes in anthers are potentially significant in research on male sterility and hybrid wheat breeding, similar reports in wheat, especially in thermo-sensitive genic male sterile (TGMS) wheat, remain elusive.

**Results:**

Herein, we systematically characterized the *formin* genes in TGMS wheat line BS366 named *TaFormins* (*TaFHs*) and predicted their functions in inducing stress response. In total, 25 *TaFH* genes were uncovered, majorly localized in 2A, 2B, and 2D chromosomes. According to the neighbor-joining (NJ) method, all TaFH proteins from wheat and other plants clustered in 6 sub-groups (A-F). The modeled 3D structures of TaFH1-A/B, TaFH2-A/B, TaFH3-A/B and TaFH3-B/D were validated. And different numbers of stress and hormone-responsive regulatory elements in their 1500 base pair promoter regions were contained in the TaFH genes copies. *TaFHs* had specific temporal and spatial expression characteristics, whereby *TaFH1*, *TaFH4*, and *TaFH5* were expressed highly in the stamen of BS366. Besides, the accumulation of *TaFHs* was remarkably lower in a low-temperature sterile condition (Nanyang) than fertile condition (Beijing), particularly at the early stamen development stage. The pollen cytoskeleton of BS366 was abnormal in the three stages under sterile and fertile environments. Furthermore, under different stress levels, *TaFHs* expression could be induced by drought, salt, abscisic acid (ABA), salicylic acid (SA), methyl jasmonate (MeJA), indole-3-acetic acid (IAA), polyethylene glycol (PEG), and low temperature. Some miRNAs, including miR167, miR1120, and miR172, interacts with *TaFH* genes; thus, we constructed an interaction network between microRNAs, *TaFHs,* phytohormone responses, and distribution of cytoskeleton to reveal the regulatory association between upstream genes of *TaFH* family members and sterile.

**Conclusions:**

Collectively, this comprehensive analysis provides novel insights into *TaFHs* and miRNA resources for wheat breeding. These findings are, therefore, valuable in understanding the mechanism of TGMS fertility conversion in wheat.

**Supplementary Information:**

The online version contains supplementary material available at 10.1186/s12864-021-07878-7.

## Background

Eukaryotic cells possess a dynamic actin cytoskeleton that controls cell growth, division, and polarity formation by alternating its global and filamentous state in response to developmental and environmental stimuli [1]. Formins (formin homology proteins), a group of proteins involved in actin polymerization, are associated with the fast-growing end (bared end) of actin filaments, potentially mediating a series of cellular functions, including polarity, division, cytokinesis, migration, among others. Several formins have been identified in plants. The formin proteins are characterized by two formin homology (FH) domains, FH1 and FH2. The FH1 (formin homology 1) domain harbors a distinct polyproline-rich region that binds to a crucial actin-binding protein-profilin or actin/profilin complex. Profilin is a vital monomer actin-binding protein and can interact with formin via the FH1 domain. The FH2 (formin homology 2) domain is characterized by a key sequence that interacts with actin [2]. Plant formins occur in two categories, type I and type II, based on the sequence homology of their FH (formin homology) domains. Type I formins have a transmembrane (TM) domain at their N-terminus followed by the C-terminal FH1 and FH2 domains, whereas type II formins do not have an N-terminal TM domain but carry an N-terminal phosphatase and tensin-related (PTEN)-like domain besides the conserved FH1 and FH2 domains [3].

Also, formins have multiple members in each species; for example, 22 formin family members are present in the Arabidopsis genome. Specifically, *AtFH1*, *AtFH5*, *AtFH6,* and *AtFH8* have been described in Arabidopsis and their functions explored in vivo. For instance, *AtFH1* overexpression in pollen tubes induced the formation of arrays of actin cables, projecting into the cytoplasm from the cell membrane. Interestingly, *AtFH1* expression induced tube broadening, growth depolarization, and growth arrest in transformed pollen tubes. With these observations, it was suggested that *AtFH1* regulation of actin polymerization could be necessary for the polarized pollen cell growth process [4]. The AtFH5-GFP fusion protein accumulated in the cell plate, essential for cell division. Furthermore, *AtFH6* regulated polarized growth by tuning the assembly of actin cables [5]. Other reports revealed that *AtFH8* affects root and root hair development by alternating actin cytoskeleton distribution [6, 7]. Collectively, formins function as key factors potentially bridging several vital pathways related to plant pollen growth and development, particularly cytoskeleton distribution. Notably, formins have been adequately characterized in animals, yeast, and Arabidopsis. For example, the first gene encoding forming― the mouse limb deformity (*ld*) gene in 1982, the mutation of which led the mice to fail to“form” proper limbs and kidneys, and a large family of formin homology proteins were related with the field of actin cytoskeleton [8, 9]. However, the *formin* gene family members are yet to be uncovered in the wheat genome. Also, it is not clear whether *formin* genes are related to pollen development, especially in the distribution of microspore cytoskeleton in wheat.

Wheat is one of the most important staple food crops in China and globally in terms of production and consumption [10]. Given that wheat production varies with climate and other environmental conditions, application of molecular biology and genetics methods is critical for advancing the stress tolerance and quality of wheat. Thermo-sensitive genic male sterile wheat (TGMS) lines, such as BS366, are of particular significance in a two-line hybrid system. The lines are highly efficient in breeding [11] because their fertility is strictly temperature-dependent [12–14]. Hybrid seeds can be produced under sterile conditions (TGMS line as the maternal plant), whereas the TGMS line can be reproduced under fertile conditions [15]. Thus, the BS366 is an ideal material for studying the genetic mechanism of TGMS and realize the effective adoption of hybrid wheat. In our previous cytological findings, the male sterility in the TGMS wheat cultivar BS366 was primarily induced by the disordered cytoskeleton distribution during meiosis of microsporocytes [16]. However, no study has reported on the contribution of *formin* gene family members related to pollen cytoskeleton distribution in TGMS wheat. Additionally, miRNAs are one type of small RNAs, which directly mediate the expression of target genes through post-transcriptional negative regulation [17]. Further information on miRNA association with the *formin* gene family is scanty. Therefore, there is a need to comprehensively explore the *formin* gene family in wheat and elucidate the interaction between *TaFHs* and miRNA; this would reveal their potential roles during anther development inBS366.

In this study, after identifying the *formin* genes of wheat (*TaFHs*), we evaluated their characteristics for the physicochemical, phylogenetic relationship, 3D structure, motif structure, exon/intro structure, chromosomal location, miRNA regulation, and expression patterns in different tissues and different development stages of anthers in the TGMS wheat line, BS366. The results presented will enrich our knowledge of the *TaFH* genes family and offer the theoretical basis and novel candidate genes for improving and developing the male sterility wheat lines. It is imperative for us to understand the fertility pathway of TGMS.

## Results

### Identifying *formin* genes in wheat

Two strategies were adopted to mine for *formin* genes in *T. aestivum* L. BLASTP using the formin of *A. thaliana* proteins as queries and HMMER searches using the formin domain as a query against the *T. aestivum* L. protein database. NCBI–CDD and SMART were used to confirm whether the formin domains were present. In total, 25 *formin* genes were identified, denoted as *TaFH1* to *TaFH10,* based on their location on the chromosomes. Based on the physicochemical analysis of the protein sequence encoded by the wheat *formin* gene, there were significant differences in physical and chemical properties across the members of the wheat *formin* family. The protein comprised 306–1403 amino acids in length, with a molecular weight of 24.40–153.70 and 5.3–9.86 isoelectric points. Subcellular localization predictions revealed that TaFH proteins functioned in cytoplasmic, nuclear, vacuolation, plasma membrane, chloroplast, and endoplasmic reticulum (Table 1). Different characteristics of *TaFH* genes and proteins were also revealed. Results demonstrated that different *TaFH* proteins exerted potentially distinct biological functions.

**Table 1:** Characteristics of the *formin* gene family members in wheat

Genes	Sequence ID	Location	length/aa	length/bp	MW(KDa)	pI	Subcellular Localization
*TaFH1-A*	TraesCS1A02G075600.1	1A: 58701490:58716429	1388	4167	151.97	7.02	cytoplasm
*TaFH1-B*	TraesCS1B02G094200.1	1B: 95651637:95653727	1403	4214	153.70	7.02	cytoplasm
*TaFH1-D*	TraesCS1D02G077900.1	1D: 59944922:59946998	1401	4206	153.21	8.09	cytoplasm
*TaFH2-A*	TraesCS2A02G388800.1	2A: 636904233:636910538	382	1149	42.62	6.42	cytoplasm
*TaFH2-B*	TraesCS2B02G406800.1	2B: 576081966:576083651	932	2799	104.47	6.01	cytoplasm
*TaFH2-D*	TraesCS2D02G386600.1	2D: 491802415:491808742	384	1155	42.85	6.12	cytoplasm
*TaFH3-A*	TraesCS2A02G183000.1	2A: 142713110:142717769	408	1227	45.75	8.95	nuclear
*TaFH3-B*	TraesCS2B02G209600.1	2B: 191747275:191752068	408	1227	45.75	8.95	nuclear
*TaFH3-D*	TraesCS2D02G190800.1	2D: 134795881:134800421	408	1227	45.75	8.95	nuclear
*TaFH4-A*	TraesCS2A02G214500.1	2A: 200622432:200625109	946	2841	101.96	7.21	vacuolation
*TaFH4-B*	TraesCS2B02G239500.1	2B: 243786328:243788946	941	2826	101.09	6.61	plasma membrane
*TaFH4-D*	TraesCS2D02G220300.1	2D: 187644176:187646539	957	2904	103.29	6.03	plasma membrane
*TaFH5-A*	TraesCS2A02G044700.1	2A: 17618132:17619862	1038	3117	107.62	7.22	chloroplast
*TaFH5-B*	TraesCS2B02G056800.1	2B: 27710139:27711846	1048	3147	108.88	6.49	chloroplast
*TaFH6-A*	TraesCS3A02G397900.1	3A: 645131154:645133017	949	2850	101.05	9.42	plasma membrane
*TaFH6-B*	TraesCS3B02G429900.1	3B: 669426515:669428377	954	2865	101.58	9.45	plasma membrane
*TaFH6-D*	TraesCS3D02G391800.1	3D: 507028317:507030177	952	2859	96.03	9.42	plasma membrane
*TaFH7-A*	TraesCS4A02G047000.1	4A: 38641338:38643658	890	2673	95.22	9.06	vacuolation
*TaFH7-D*	TraesCS4D02G257700.1	4D: 427461005:427463346	886	2661	94.82	9.06	plasma membrane
*TaFH8-A*	TraesCS5A02G075500.1	5A: 90913931:90925317	306	921	34.40	5.3	chloroplast
*TaFH9-B*	TraesCS6B02G389300.1	6B: 664230386:664232873	1336	4011	145.50	6.37	chloroplast
*TaFH9-D*	TraesCS6D02G339300.1	6D: 438905707:438908215	1335	4008	145.46	6.5	endoplasmic reticulum
*TaFH10-A*	TraesCS6A02G312000.1	6A: 548402427:548404312	829	2490	86.92	9.86	chloroplast
*TaFH10-B*	TraesCS6B02G342200.1	6B: 602822952:602824912	901	2706	94.01	9.72	chloroplast
*TaFH10-D*	TraesCS6D02G291400.1	6D: 402117660:402119621	901	2706	94.22	9.72	chloroplast

### Phylogenetic tree and domain analysis of TaFHs

To elucidate the phylogeny of formin proteins, we adopted the MEGA software to analyze the 25 identified *formin* genes using an evolutionary tree. Based on the information on the phylogenetic relationship, 25 *TaFH* genes were clustered into 10 groups (Fig. S1). Results demonstrated that 25 *TaFH* genes could be classified into four groups; the large group could then be subdivided into four groups. The conservative domains of these domain were the regions where genes function. The TaFHs structure were obtained and the number of exons and introns were highly divergent (Fig. S2). Most members of TaFHs shared the similar patterns of exon/intro structure, including intro phase, intro number and exon length.

**Fig. 1:**
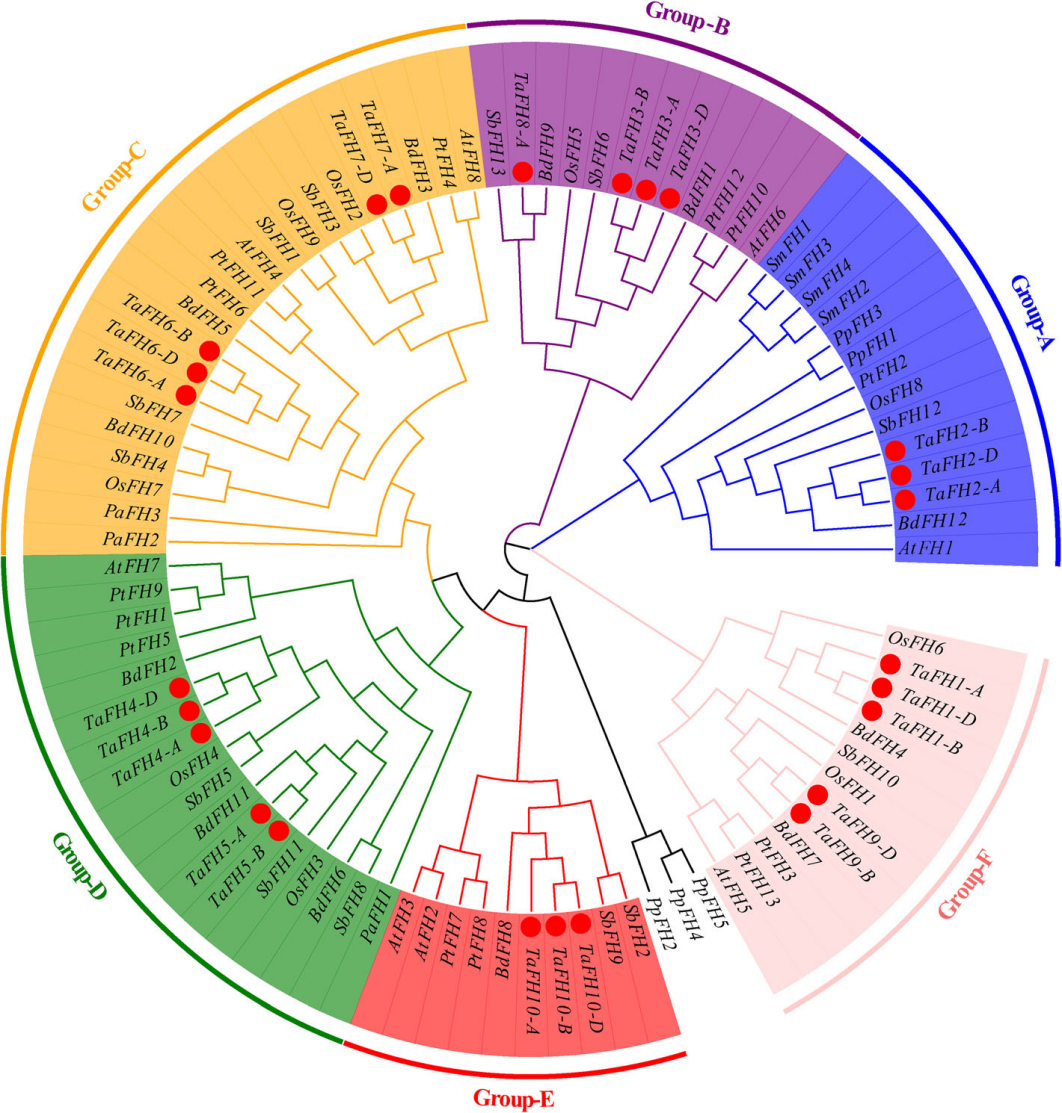
The phylogenetic tree of TaFH and other species proteins. The tree was constructed from a complete alignment of formins from* T. aestivum *(Ta), *P. patens* (Pp), *S. moellendorffii *(Sm), *P.trichocarpa* (Pt), *S. bicolor *(Sb), *B. distachyon* (Bd), *A.thaliana* (At), and *O. sativa *(Os), then using MEGA 6.0 by the neighbor-joining method with 1000 bootstrap replicates; bootstrap scores are depicted on the nodes

To reveal the functional information of *TaFH* genes, we constructed a phylogenetic tree based on the comparison among the *T. aestivum*, *P. patens*, *S. moellendorffii*, *P. trichocarpa*, *S. bicolor*, *B. distachyon*, *A. thaliana*, and *O. sativa* (Fig. 1). All the formin proteins clustered into six groups, except for the PpFH2, PpFH4 and PpFH5. In total, 25 sequences of 10 members of the wheat *formin* gene family clustered into six subgroups. Moreover, we found that the TaFH proteins were clustered into the same clades with some BdFH, SbFH and OsFH proteins. For example, TaFH2, BdFH12, SbFH12 and OsFH8 were clustered into group A, BdFH1, SbFH6 and OsFH5 were clustered with TaFH3 on the group B, TaFH4 and TaFH5 were clustered into the group D with BdFH2, BdFH11, SbFH5, SbFH11, OsFH4 and OsFH3, and TaFH9 were clustered into the group E with BdFH7, SbFH10 and OsFH1. These showed that TaFH proteins had a close correlation with those in *B. distachyon*, *S. bicolor* and *O. sativa* and shared a low similarity with those in *P. patens*.

In this study, eight TaFHs representative proteins were modeled using homology to predict the 3D structure. The Phyre 2 server and PyMDLWin been used to deduced and visualized, which were shown in Fig. S3. The results also shown that protein structure of A, B and D homoeologous copies with TaFH1-A/B, TaFH2-A/B, TaFH3-A/B and TaFH9-B/D had shown significant similarity. The 3D structure of TaFH proteins laid a foundation to explor the complex mechanism of formins at the structure level.

### Chromosomal assignment of *TaFH* genes

**Fig. 2:**
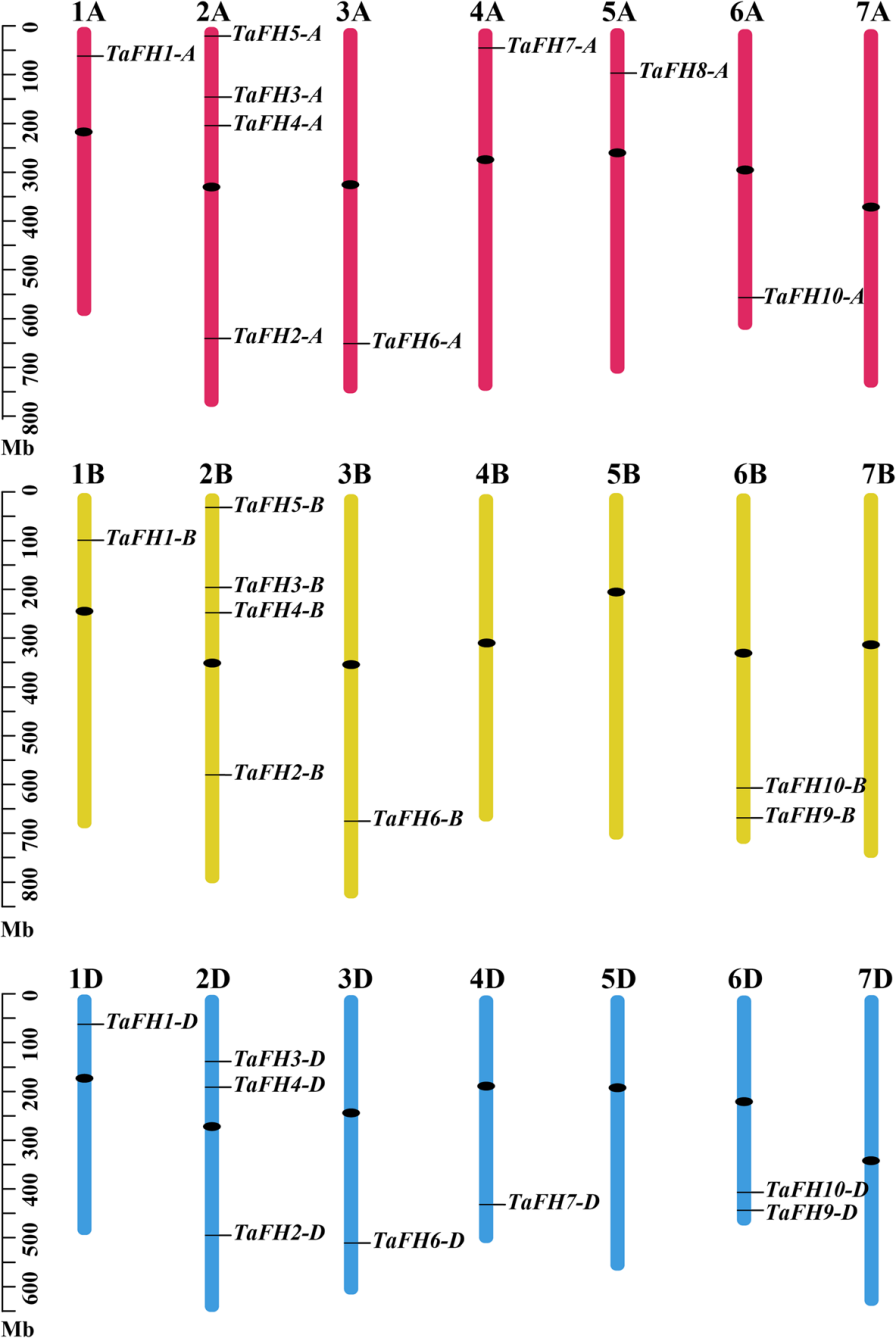
Chromosomal assignment of the *formin* gene family in wheat. The chromosome numbers are indicated at the top of each bar, whereas the size of a chromosome is denoted by the unit of the left scale with Mb. Black dots on the chromosome denotes the position of the centromeres

Map files were constructed based on the location of the wheat *formin* gene family on the chromosome. A chromosome distribution map was drawn using the MapInspect software. A total of 25 wheat *formin* genes were located on the chromosomes (Fig. 2). Three copies of *TaFH1*, *TaFH2*, *TaFH3*, *TaFH4*, *TaFH6,* and *TaFH10* were located on sub-genomes A, B, and D of wheat chromosomes 1, 2, 3, and 6. Two copies of *TaFH5*, *TaFH7,* and *TaFH9* were located on sub-genomes of wheat chromosomes 2, 4, and 6, respectively. *TaFH8* had one copy on the short arm of chromosome 5A. Four *formin* genes were found on chromosomes 2A and 2B, and three *formin* genes on chromosome 2C, existing in clusters. However, no *formin* gene was present on 7A, 4B, 5B, 7B, 5D, and 7D, and this demonstrated a widespread but uneven distribution of the wheat *formin* genes on the wheat chromosome.

### MicroRNA targeting prediction of *TaFH* genes

**Fig. 3:**
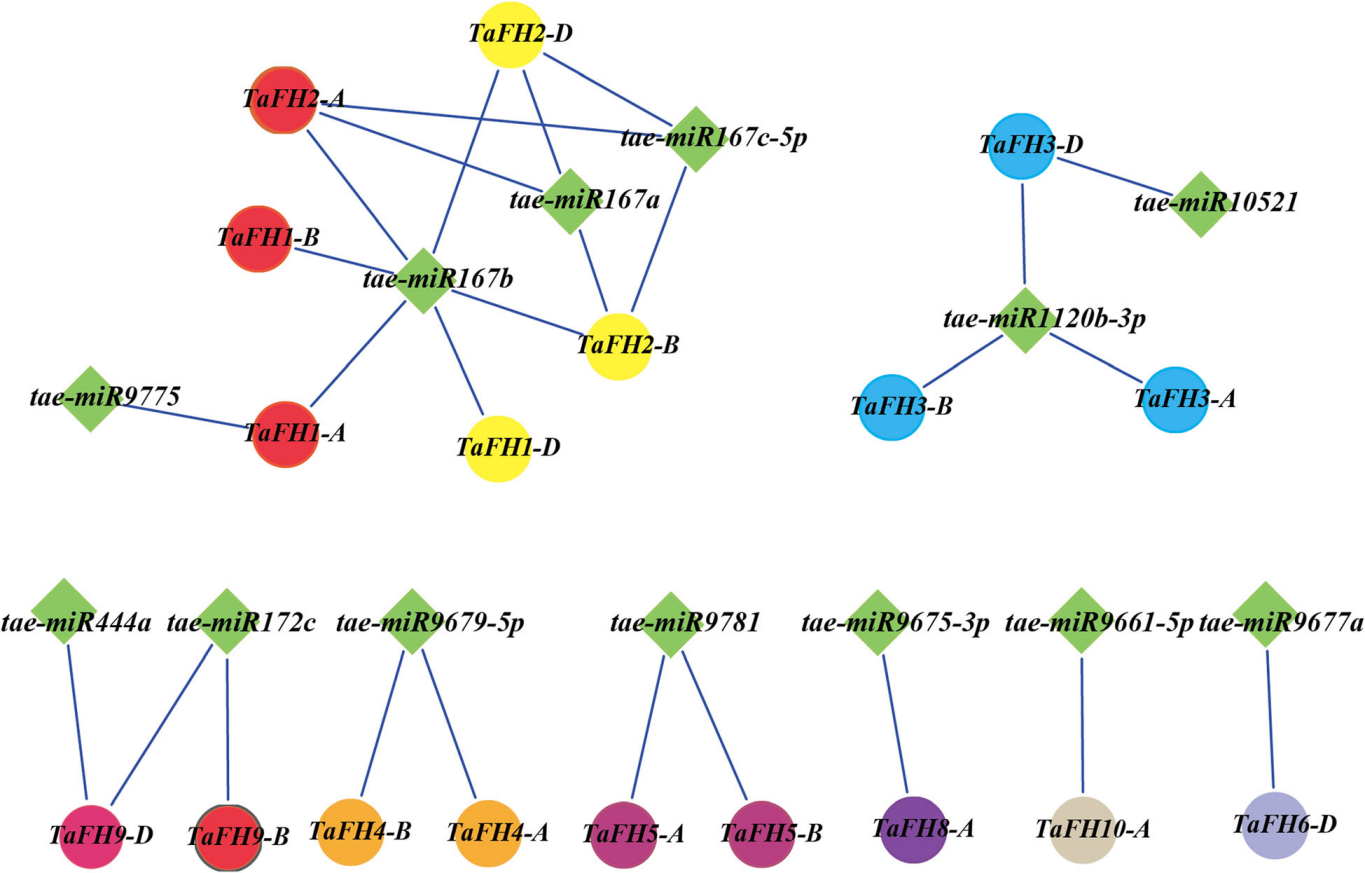
The relationship analysis of miRNAs and their target genes in *formin* genes in wheat

To further understand the function of *TaFH* genes, we evaluated the binding association of the *TaFH* genes with miRNAs. Results revealed that 19 of the 25 *TaFH* genes harbored targets for 13 different miRNAs (Fig. 3 and Table S1). We also found that tae-miR167a, tae-miR167b, and tae-miR167c-5p potentially target *TaFH2-A*, *TaFH2-B,* and *TaFH2-D*. Indeed, Tae-miR167 has been shown to target other wheat genes. For example, tae-miR167a directly targeted Auxin Response Factor 8 (*TaARF8*), which possibly regulated auxin biosynthesis by impeding the downstream genes. Also, tae-miR167a overexpression in Arabidopsis lowered the expression levels of its targets (*AtARF6* and *AtARF8*) and induced male sterility phenotypes [18]. Our results suggested that tae-miR167b might target *TaFH1-A*, *TaFH1-B,* and *TaFH1-D*. Tae-miR1120b-3p has been shown to target *TaFH3-A* and *TaFH3-B*, and possibly *LRR*, thereby regulating the jasmonate biosynthesis or auxin response pathway associated with pollen development. A previous study found that the *Q* gene, involved in bread wheat spike architecture, was suppressed by tae-miR172 [19]. In a separate study, Tae-miR444a targeted *TaFH9-D* and regulated floral patterning and development via the transcription factor MADS-box [20]. Collectively, the findings of this study could help to further understand the regulation between miRNA and *formin* in the related TGMS pathway.

### Promoter analysis of *TaFH* genes

**Fig. 4:**
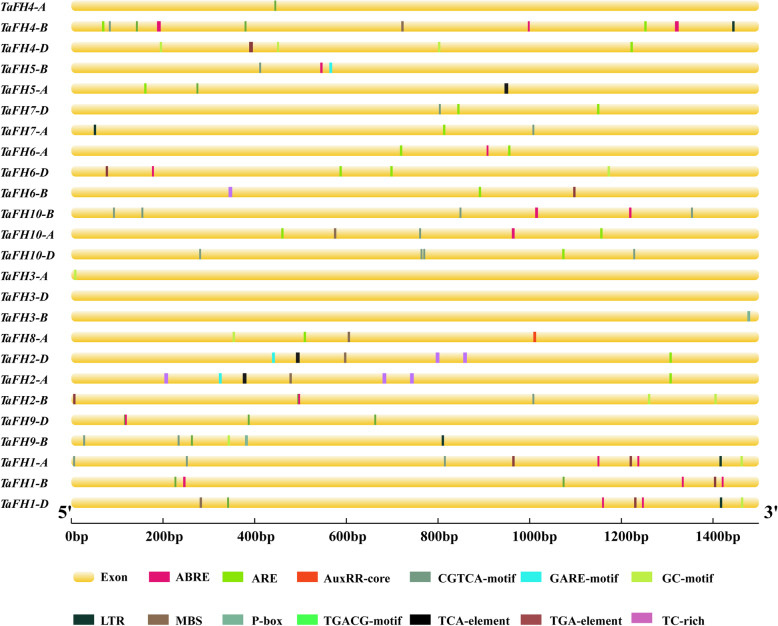
Analysis of specific *cis*-elements in promoters of *TaFHs*. The 1.5 k-bp promoter sequences of corresponding *TaFH* genes were used to analyze specific hormone-related *cis*-elements and stress-responsive regulatory elements, which are color-coded. ABRE: abscisic acid responsive element; ARE: antioxidant response element mediating transcriptional activation of genes exposed to oxidative stress; AuxRR-core: auxin-responsive element; CGTCA-motif and TGACG-motif: MeJA-responsive element; GARE-motif and P-box: gibberellin-responsive element; GC-motif: enhancer-like element involved in anoxic specific inducibility; LTR: low-temperature responsive element; MBS: drought-inducible element; TCA-element: salicylic acid responsive element; TGA-element: auxin responsive element; TC-rich repeat: defense and stress responsive element

To establish how the expression levels of *TaFH* genes responded to stress stimuli, 1.5-kb upstream promoter regions of *TaFH* genes were scanned for stress-related *cis*-regulatory elements using the Plant CARE online service. Eight hormone-responsive regulatory elements (ABRE, TGA-element, AuxRR-core, TGACG-motif, CGTCA-motif, P-box, GARE-motif, and TCA-element) associated with ABA, auxin (IAA), methyl jasmonate (MeJA), gibberellin (GA), and salicylic acid (SA) responses were identified in the promoter region of *TaFHs* (Fig. 4) Additionally, five stress-responsive regulatory elements (TC-rich repeats, MBS, ARE, GC-motif, and LTR) associated with defense/stress and low-temperature responses were identified in the TaFH promoter regions. Different types and numbers of regulatory elements were present in the distinct TaFH promoters. This implied that *TaFH* genes are potentially associated with the response to various stress and hormone treatments by mediating different regulatory mechanisms.

### Tissue/organ-specific expression profiles of *TaFH* genes

**Fig. 5:**
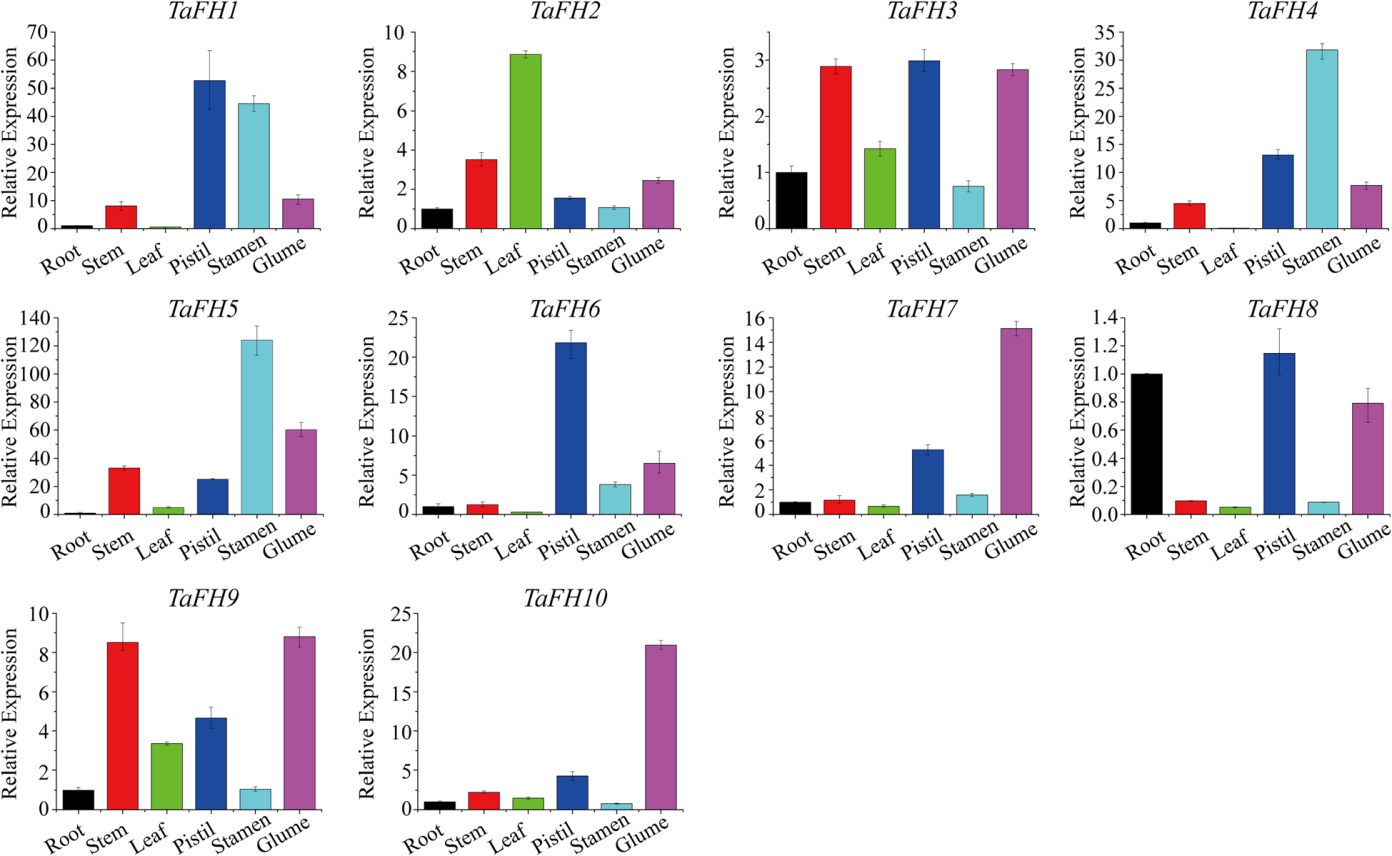
The tissue expression patterns of the *formin* gene family in wheat, including root, stem, leaf, pistil, stamen, and glume. Different colors denoted different tissues; black for root, red for the stem, green for leaf, mazarine for pistil, wathet for stamen, purple for glume. The expression in root was set as 1. The error bars represent the standard error of the mean

We explored the expression of *TaFH* genes in different tissues of TGMS wheat line BS366, including the root, stem, leaf, pistil, stamen, and glume. All the *TaFH* genes were constitutively expressed in all the tissues. The expression of *TaFH1* in pistils was the highest, whereas that in stamens was about 45 folds more than in the roots (Fig. 5). The expression of *TaFH6*, *TaFH3*, and *TaFH8* in pistils was markedly higher than those in other tissues, about 20, 3 and 1.2 folds more than in roots. The expression of *TaFH4* and *TaFH5* in stamens was the highest, about 30 and 120 folds more than that in roots, implying that *TaFH1*, *TaFH4* and *TaFH5,* were potentially linked to anther development. Also, *TaFH2* expression in leaves was the highest, nearly 9 folds higher than in the roots, but relatively lower in the corresponding tissues of flower organs. This suggested that *TaFH2* could be mainly related to the development of leaves. The expression of *TaFH7* in glume was the highest, nearly 16 folds higher than that in the roots. The expression levels of *TaFH9* and *TaFH10* in glumes were higher than the root, stem, leaf, pistil and stamen, about 9 and 20 folds than in roots, respectively. Thus, we speculated that *TaFH7*, *TaFH9,* and *TaFH10* could be related to the opening and closing of glumes. Further analysis revealed that 10 members of the wheat *formin* gene family were mainly associated with the development of the above-ground tissues of BS366, mostly the floral organs. In particular, they contributed to the development of anthers and glumes, and other metabolic processes in TGMS wheat.

### Analysis of *TaFH* genes family expression patterns in fertility sensitive stages under different fertility conditions in BS366

**Fig. 6:**
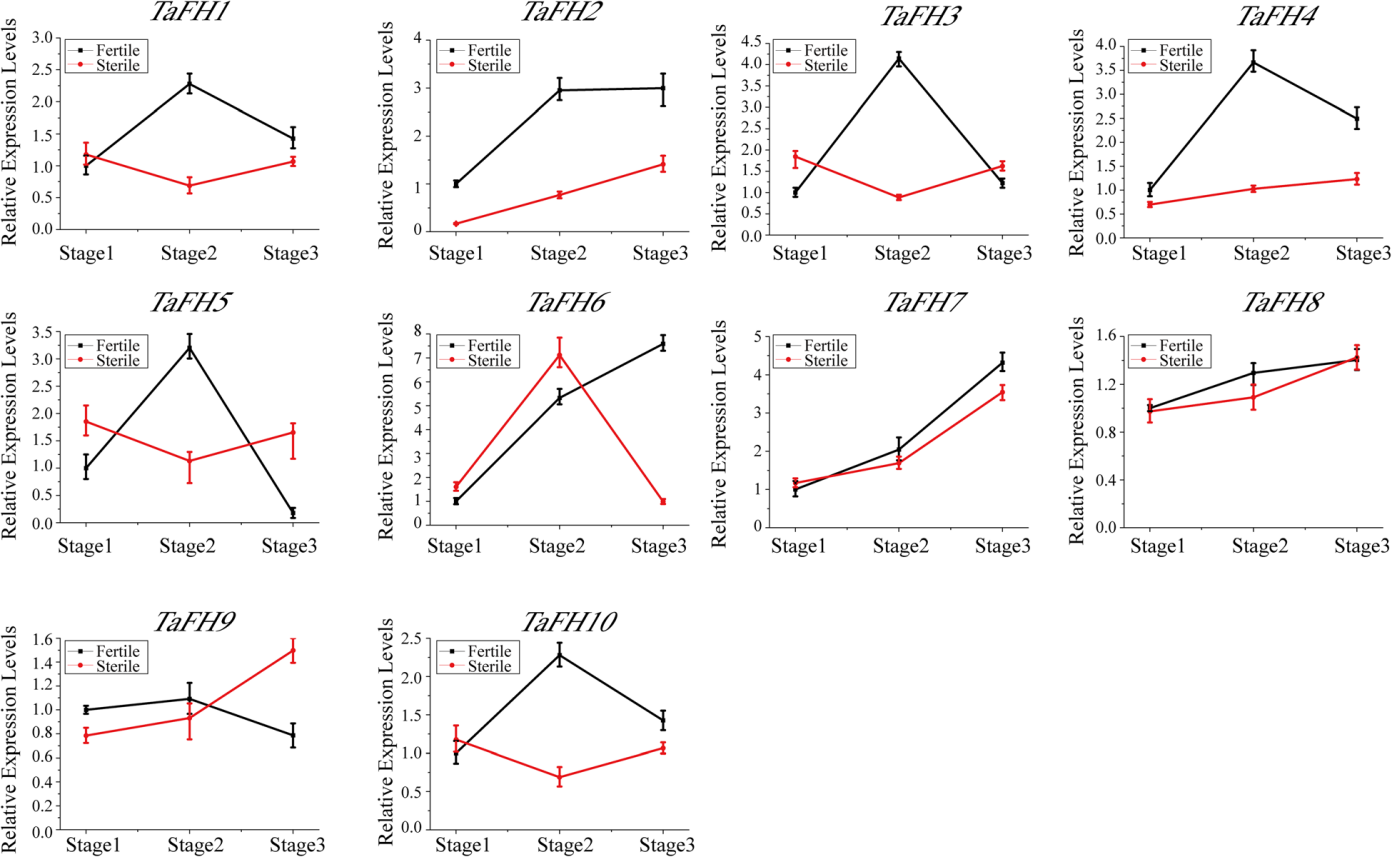
Expression profiling of *TaFH* genes at three anther developmental stages in fertile and sterile conditions. Fertile condition: 20 °C with 12-h day/12-h night for daily mean temperature during pollen development stages; sterile conditions: 10 °C with 12-h day/12-h night for daily mean temperature during pollen development stages. Stage 1: secondary sporogenous cells had formed, stage 2: all cell layers were present, and mitosis had ceased, and stage 3: meiotic division stage, respectively. The error bars represent the mean ± standard deviation of three replicates

Based on previous reports, the fertility sensitive stage of BS366 was the meiosis stage of microspore mother cells [10, 16]. Herein, we selected three key meiotic stages (1, 2, 3) for expression pattern analysis. The role of the wheat *formin* gene family in pollen fertility was further elucidated by analyzing the expression of *TaFHs* in the three stages of stamen development in BS366 under different fertility environments (fertile and sterile conditions). The expression levels of *TaFH2* and *TaFH4* in the corresponding three stages of stamen development under sterile conditions were lower than those under fertile conditions (Fig. 6). *TaFH1* expression was higher at the early stage (stage 1) under sterile conditions. The expression of *TaFH1*, *TaFH3*, *TaFH5,* and *TaFH10* were significantly higher at stage1, but stage 2 under sterile conditions. On the other hand, *TaFH6* expression was higher at the early stages of stamen development (stage 1 and 2) under sterile conditions. *TaFH7* and *TaFH8* showed no obvious expression changes at the three stages under both fertility and sterile conditions. However, *TaFH9* expression was lower under sterility conditions than in fertility conditions at stage 1 and 2. These results implied a potential association of *TaFH* genes with stamen development and fertility transformation via particular pathways.

### Distribution of cytoskeleton in meiosis of microsporocytes

**Fig. 7:**
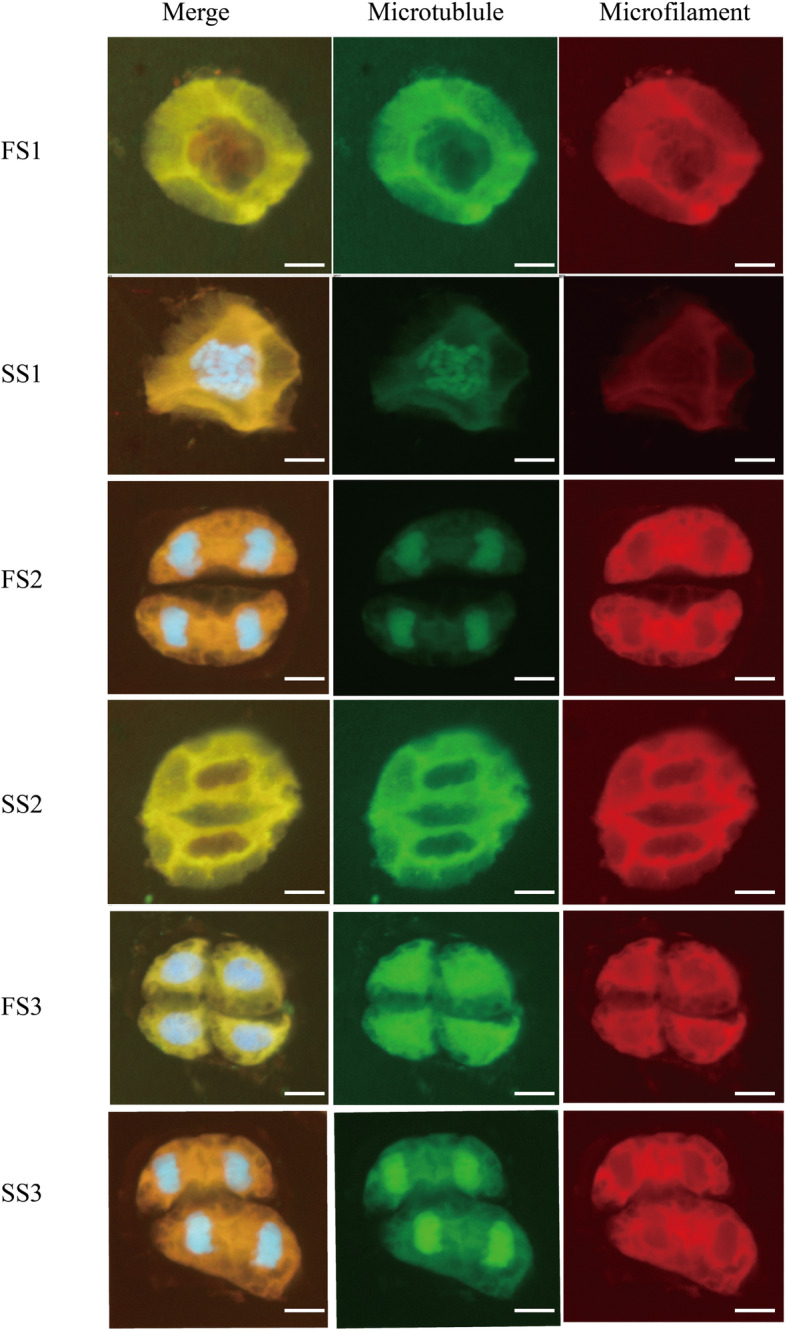
The distribution of cytoskeleton and chromatin in pollen cells of a genic male sterile wheat line BS366 during the meiosis stage. The TGMS plants in sterile (SS) and fertile condition (FS) of different stage, respectively. The number 1, 2, 3 indicated stage 1: secondary sporogenous cells had formed; stage 2: all cells layers were present and mitosis had ceased, and stage 3: meiotic division stage, respectively. Bars = 10 μm

Compelling evidence shows that the pollen cell cytoskeleton of BS366 is abnormal at low temperature, and the *formin* gene is highly critical in the development of the pollen cell cytoskeleton [15, 16]. In the present study, to establish any change in the cytoskeleton of the TGMS 366 in the fertility and sterility environment, the pollen cell was evaluated in three key meiotic stages. We found abnormal cytoskeleton distribution in male-sterile wheat pollen cells (Fig. 7). The pollen cell shrank, and the cytoskeleton was loose and disarranged in stage 1. The cell plate was absent, and the microtubule and microfilaments nonpolar terminus were vague in stage 2. In stage 3, the microtubule and microfilaments disappeared. Collectively, these findings suggested that differential expression of *TaFH genes* might cause the abnormal pollen cytoskeleton of BS366 under low temperatures.

### Expression profiles of *TaFH* genes under different exogenous phytohormone treatments and abiotic stress

**Fig. 8:**
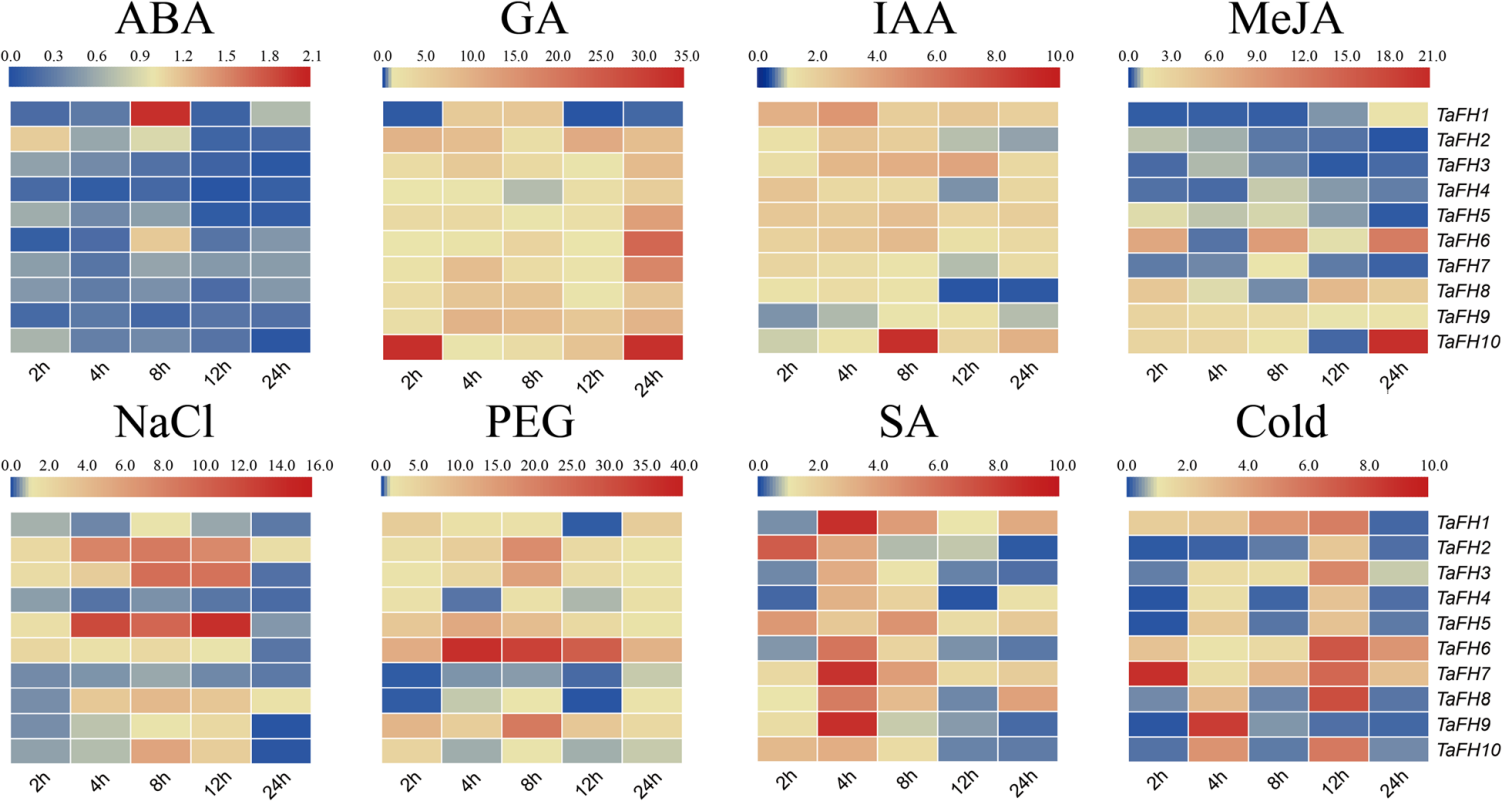
Stress expression analysis of *formin* gene family in TGMS wheat line BS366 under 5 phytohormones (ABA, GA, IAA, MeJA, SA), NaCl, PEG, and cold treatments. The expression at 0 h was set as 1 (data not shown)

Abscisic acid (ABA), gibberellin (GA), indole-3-acetic acid (IAA), methyl jasmonate (MeJA), and salicylic acid (SA) are the main plant growth regulators that exert critical functions in plant growth and metabolism. By exploring the *cis*-element of TaFH, we found that TaFH had different types and numbers of regulatory elements. To affirm the function of *TaFH*, we conducted a qRT-PCR assay to examine the expression pattern of the *formin* genes in the leaves of BS366 following different hormone treatments (Fig. 8).

According to the results, all the *formin* genes slightly responded to the ABA, GA, IAA, MeJA, and SA treatments. Specifically, *TaFH3*, *TaFH4*, *TaFH5*, *TaFH7*, *TaFH8*, *TaFH9,* and *TaFH10* were no obvious expressed changing under abscisic acid-induced stress, the expression of *TaFH1* and *TaFH6* was initially inhibited but later rose and peaked at 8 h, after which it began to show a wave-like decreasing trend and then rose again. Under auxin (IAA) stress, *TaFH1*, *TaFH2*, *TaFH3*, *TaFH4*, *TaFH5*, *TaFH6*, *TaFH7*, *TaFH8,* and *TaFH10* were initially overexpressed, but their expression later decreased. The expression of *TaFH1*, *TaFH2,* and *TaFH8* peaked at 4 h, whereas *TaFH9* expression was impeded and then increased, and later peaked at 12 h. The expression of *TaFH2*, *TaFH3*, *TaFH4*, *TaFH5*, *TaFH6*, *TaFH7*, *TaFH8*, *TaFH9,* and *TaFH10* demonstrated a certain upward trend under gibberellin stress. The expressions of *TaFH2* and *TaFH10* rose significantly at 2 h after gibberellin stress, then decreased and increased again. *TaFH3*, *TaFH4*, *TaFH5*, *TaFH6*, *TaFH7*, *TaFH8,* and *TaFH9* showed a wave-like increasing trend, then decreased and rose again. The expression of *TaFH4*, *TaFH5*, *TaFH6, TaFH7,* and *TaFH8* peaked at 24 h, whereas that of *TaFH9* peaked at 4 h. Under methyl jasmonate stress, *TaFH1* expression was inhibited but later increased at 12–24 h. The expression of *TaFH2*, *TaFH3*, *TaFH4,* and *TaFH5* was inhibited within 24 h, and that of *TaFH6* and *TaFH10* showed an upward and downward trend, then peaked at 24 h. Further, the expression of *TaFH7* was inhibited at 2 h, then increased and later decreased. *TaFH8* expression peaked at 8 h, increased at 2 h, but then decreased, increased, and then decreased again at 12 h; the relative expression peaked at 2 h. The expression of *TaFH9* was enhanced, then inhibited, and peaked at 8 h. Under salicylic acid stress, the relative expression of *TaFH2*, *TaFH5*, *TaFH7*, *TaFH8*, *TaFH9,* and *TaFH10* was higher at 2 h compared to 0 h. *TaFH2* expression peaked at 2 h, while *TaFH5* peaked at 8 h. The expression of *TaFH7*, *TaFH8*, *TaFH9* and *TaFH10* all peaked at 4 h. Besides, the relative expression of *TaFH1*, *TaFH3* and *TaFH6* decreased at 2 h, whereas that of *TaFH1* decreased at 8 h. Also, the expression of *TaFH3*, *TaFH4,* and *TaFH6* peaked at 4 h. These results demonstrated that *formin* genes might be central to hormone regulation in the BS366 wheat.

To evaluate the response mechanism of the *formin* gene family to cold stress, drought, and high salt, we employed qRT-PCR to analyze the expression pattern of the *formin* genes in the leaves of BS366 under low temperature (10 °C), salt (NaCl), and drought stress (PEG) treatments.

Initially, the expression of *TaFH2*, *TaFH3*, *TaFH4*, *TaFH5*, *TaFH8*, *TaFH9,* and *TaFH10* was inhibited under cold stimulation, but later rose. The expression of most *TaFH* genes peaked at 12 h; however, the expression of *TaFH1*, *TaFH6,* and *TaFH7* rose and then decreased post-cold stimulation. High salt stress impeded the expression of *TaFH1*, *TaFH4*, *TaFH7*, *TaFH8*, *TaFH9,* and *TaFH10*, whereas the expression of *TaFH2*, *TaFH3*, *TaFH5,* and *TaFH6* showed an opposite trend. Under abiotic drought stress, except for *TaFH7* and *TaFH8*, the expression of *TaFH2*, *TaFH3,* and *TaFH9* peaked at 8 h and then decreased. These results indicate that *TaFH* genes might respond to different plant hormones and participate in a complex regulatory network in thermo-sensitive male sterile line BS366.

## Discussion

### The relationship between *formin* genes and cytoskeleton in wheat

Formins initiate actin assembly and control key cytoskeleton genes. They regulate the nucleation of actin and mediate *profilin* and *Arp2/3* during cell division. Also, they participate in vegetative and reproductive plant growth [21]. Previously, [15] found abnormal cytoskeleton formation in a TGMS line under sterile environment. In this study, we examined the cytoskeleton of the pollen-sensitive stages in BS366 using scanning electron microscopy and found aberrant nuclear division in the pollen cells. Real-time quantification analysis of the *formin* genes revealed different expression of *TaFH1*, *TaFH2*, *TaFH3*, *TaFH4*, *TaFH5*, *TaFH6, TaFH9* and *TaFH10* under sterile and fertile conditions at these stages. Therefore, we concluded that the *formin* genes *TaFH1*, *TaFH2*, *TaFH3*, *TaFH4*, *TaFH5*, *TaFH6*, *TaFH9,* and *TaFH10* might affect the cytoskeleton, resulting in abnormalities during meiosis and thus male sterility in TGMS BS366.

### Effect of microRNA regulation via *formin* genes on the fertility of TGMS line

MiRNAs are endogenous, small, non-coding RNAs, exerting critical roles in the regulation of hormone signaling pathways and cellular functions during developmental processes [22]. Of note, miRNAs participate in the regulation of male fertility in crops [23]. Particularly, miR167 plays a dominant role in plant development. In Arabidopsis, miR167 directly targets *ARF6* and *ARF8*, which regulates jasmonate biosynthesis by impeding downstream genes, thereby affecting pollen development. In rice, miR167 potentially targets *ARF6*, *8*, *12*, *17,* and *25* genes*.* Notably, *ARF8* can interact with *OsGH3–2.* Besides, OsmiR167 responds to exogenous auxin via its target gene *OsARF8*. Abnormal *OsGH3–2* expression induces morphological aberrations, including short plants and small panicles [24–26]. Studies have shown that TaemiR167a, TaemiR167b, and TaemiR167d are abnormally expressed in the sensitive stage of pollen in wheat line BS366 [27, 28]. TaemiR167a targeted *AtARF6* and *AtARF8* upon its translation into Arabidopsis*,* and influenced the synthesis of IAA and JA, thereby regulating the development of anthers [18].

In the present study, by analysis the relationship between the miRNA and *formins*, we found that taemiR167a targeted *TaFH2-A*, *TaFH2-B*, and *TaFH2-D;* taemiR167b targeted *TaFH1-A*, *TaFH1-B*, *TaFH1-D*, *TaFH2-A*, *TaFH2-B,* and *TaFH2-D;* whereas taemiR167c-5p targeted *TaFH2-A*, *TaFH2-B,* and *TaFH2-D*. As such, we suggested that taemiR167 might regulate the *formin* genes in pollen-sensitive stages and influence BS366 fertility; leading to abnormal anther dehiscence. Moreover, the gene FBA (fructose-1, 6-bisphosphate aldolase) was predicted to be the target of miR1120. Of note, miR1120 could be regulated by ABA under low-temperature treatment, which affected the expression of *FBA*, then regulated the sugar metabolism to mediate ATP generation [28]. In a previous report, the *OsFH5* mutants were characterized by aberrant inflorescence. The cytological analysis demonstrated that *OsFH5* mutants exhibited severe cell elongation defects and abnormal microtubule arrays [29]. Herein, miR1120 was predicted to target *TaFH3* (Fig. 3), *OsFH5,* and *TaFH3,* clustered into group B (Fig. 1). Also, the cytoskeleton was abnormal during anther development. Thus, we speculated that miR1120 potentially regulated the energy synthesis and then impacted the pollen cell division and the fertility of the TGMS line BS366 under low-temperature treatment.

### Expression and functions of *formin* genes in response to abiotic stressors

A previous study by [27] found abnormal levels of IAA during TGMS366 pollen development. Other reports have also revealed that MeJA, JA, and SA influence the pollen spill out, which results in the sterility of BS366 [30, 31]. In this study, we isolated the *formin* gene family, including 25 members from wheat (Table 1). The *formin* genes exhibited different expression levels in different BS366 tissues. For instance, the relative expression of *TaFH4* and *TaFH5* in stamen was higher compared to other tissues. Also, the relative expression of *TaFH1*, *TaFH3*, *TaFH6,* and *TaFH8* in pistil was higher than in other tissues, suggesting that *TaFH1*, *TaFH3*, *TaFH4*, *TaFH5, TaFH6,* and *TaFH8* potentially contribute to the sterility of BS366. Furthermore, the relative expression of *TaFH1*, *TaFH2*, *TaFH3*, *TaFH4*, *TaFH5, TaFH6*, *TaFH9,* and *TaFH10* was different in the sensitive stage between the fertility and sterility conditions, implying that they might play a regulatory role in wheat fertility.

*Cis*-regulatory sequences, such as enhancers and promoters, influence development and physiology by regulating gene expression [32]. *Cis*-regulatory elements, functioning as important molecular switches, are associated with the regulation of gene transcription under external stimuli [33]. To comprehensively elucidate the functions of *TaFH* genes, we assessed *cis*-acting regulatory elements and expression profiles under various stresses. Eight responsive regulatory elements and five stress-responsive regulatory elements were identified in the *TaFH* promoter region. These findings demonstrated that the *TaFH* genes respond to stress and hormone treatment variations via different mechanisms. The *TaFHs* contained different types and numbers of *cis*-acting regulatory elements in each promoter region. These genes, therefore, were characterized by different regulatory functions that responded to variations in stress and hormone treatments. We also evaluated the expression profiles of *TaFH* genes in TGMS wheat line BS366 seedlings under different stress conditions and hormone treatments. The expression profile analysis revealed that TaFH8 and TaFH10 had similarly structured proteins upregulated by the auxin response treatment. Similar expression profiles were shown in the *TaFH2* following low-temperature treatment.

Moreover, the response of *formin* genes to the ABA, NaCl, and PEG implied that *TaFHs* might exert multiple functions in plants, including roles in abiotic stress resistance and pollen fertility. Although *formin* is a key regulatory gene in the cell cytoskeleton, exploring the mechanisms by which it responds to low temperature, affects the cytoskeleton, and defines pollen development of wheat would be vital to reveal the actual function of *TaFH*.

### Possible miRNA-TaFH interaction network in wheat

**Fig. 9:**
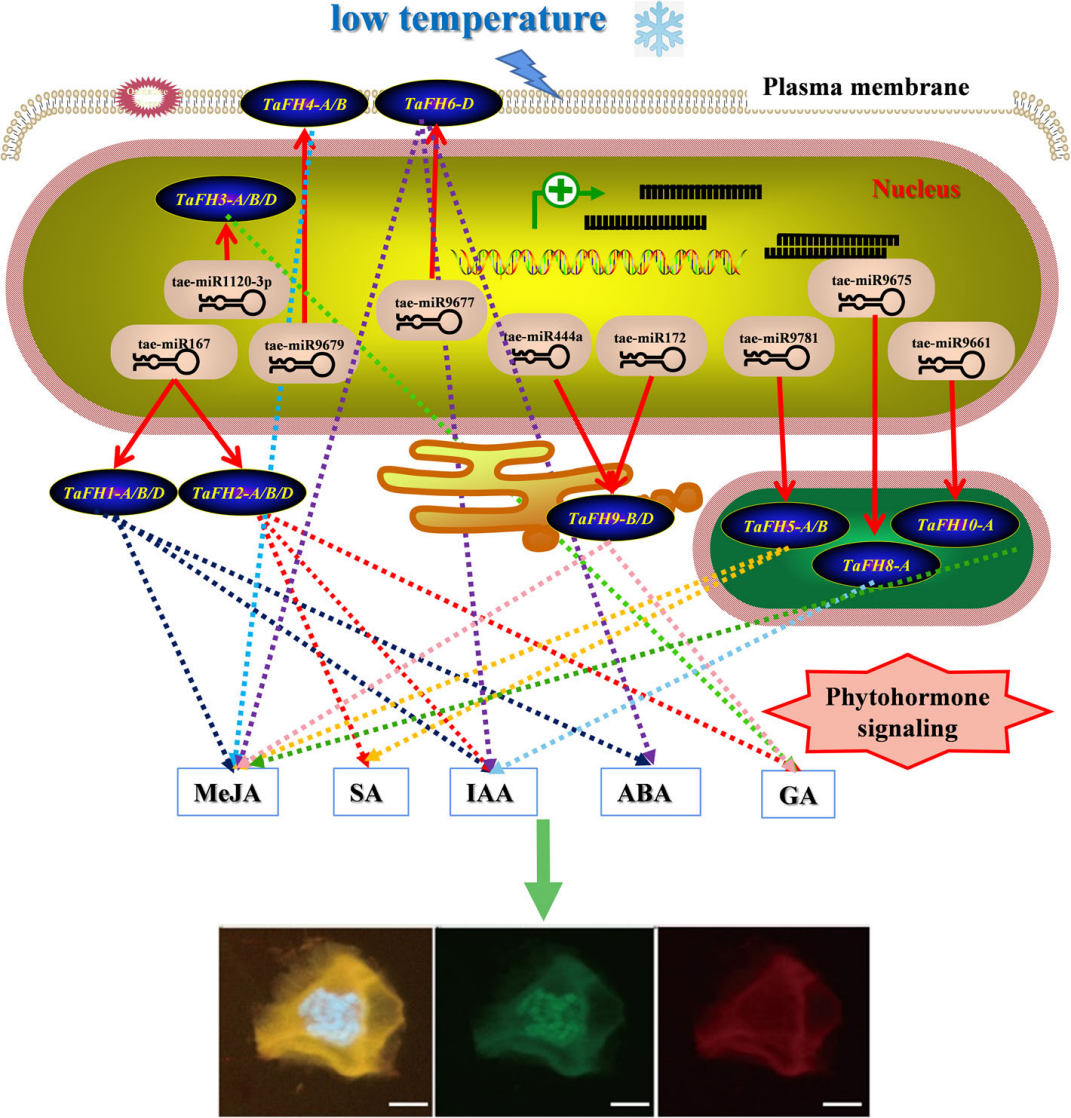
Putative regulatory network of miRNAs and targeted *formin* genes in wheat. Arrows with dotted lines indicate the most sensitive responses to stresses of each *TaFH* gene. Arrows with a solid line indicate the interactions of tae-miRNAs and target *TaFH* genes negative control. Tae-miR167 may target *TaFH1* and *TaFH2* in the cytoplasm; tae-miR1120-3p may target *TaFH3* in the nucleus; tae-miR9677 and tae-miR9679 may target *TaFH6* and *TaFH4* in the plasma membrane; tae-miR444a/tae-miR172 may targeted *TaFH9* in endoplasmic reticulum; tae-miR9781, taemiR9675, and tae-miR9661 may target *TaFH5*, *TaFH8*, and *TaFH10* in chloroplast

In this study, we proposed a potential regulatory pathway of *TaFH* (Fig. 9). The functions of the *TaFH* gene family were established by multiple endogenous and exogenous factors, and the regulatory network was complex. Therefore, we speculated that these miRNAs of tae-miR167, tae-miR1120-3p, tae-miR9677, tae-miR9679, tae-miR444a, tae-miR172, tae-miR9781, taemiR9675 and tae-miR9661 could inhibit the expression of target *TaFHs* at the transcriptional or translational levels under low-temperature conditions. This regulation strategy could have contributed to the abnormal cytoskeleton and the meiosis abnormalities, resulting in abnormal pollen. Besides, *TaFH* genes are associated with the signaling pathway of MeJA, SA, IAA, ABA, and GA. In this study, we found that *TaFH1* and *TaFH2* were regulated by IAA, *TaFH4* and *TaFH6* could respond to MeJA, and *TaFH3* and *TaFH9* were mainly regulated by GA. Collectively, these findings suggested that *TaFH1*, *TaFH2*, *TaFH3,* and *TaFH9* might play a crucial role in the regulation of temperature-induced male sterility in wheat TGMS line BS366.

## Conclusions

In this study, we systematically uncovered *TaFH* genes in the wheat genome. A total of 25 *TaFHs* were identified, each containing a conserved FH2 domain. The chromosome locations, gene and protein structures, subcellular localization, phylogenetic relationships, Tae-miR167, Tae-miR1120, Tae-miR172, and Tae-miR444 binding sites, and *cis*-elements were also characterized. The *TaFH* expression levels in different tissues demonstrate their potential role in flower development. The pollen cytoskeleton of BS366 was abnormal in the three stages under fertility and sterility conditions. Based on quantitative RT-PCR analysis, the tested *TaFH* genes were highly expressed in inflorescences and response to abiotic stressors. Collectively, these findings establish a foundation for further exploration of *TaFH* genes and provide novel insights into their biological functions.

## Methods

### Sources of sequence data

The whole-genome sequences of *Triticum aestivum* L. (*T. aestivum*, Ta) were retrieved from the wheat genome Ensemble database (http://plant.ensembl.org, IWGSC RefSeqv1.1). To identify members of the *formin* genes family from *T. aestivum*, we adopted two methods: Firstly, the hidden Markov model (HMM) profile of the formin (accession number in Pfam: PF06507) was downloaded from the Pfam database (http://pfam.sanger.ac.uk/) [34] and used as a query to search for candidate formin proteins in the *T. aestivum* proteome using HMMER3.0 [35]. Additionally, Arabidopsis formin protein sequences were adopted as queries to reveal the *T. aestivum* L. proteome via BLAST. Genes collected from the above two methods were integrated, and duplicates were eliminated manually. All protein sequences of the candidate *formin* genes were verified using domain analysis programs NCBI–CDD (https://www.ncbi.nlm.nih.gov/cdd/) and SMART (Simple Modular Architecture Research Tool; http://smart.emblheidelberg.de/), the gene structure with intro-exon boundaries of *TaFH* gene family using the Gene Structure Display Server (GSDS). Basic physical and chemical parameters of TaFH proteins, including amino acid length, theoretical isoelectric point, and molecular weight, were obtained from an online program, ProtParam (https://web.expasy.org/protparam/).

### Phylogenetic analysis

The formin coding sequences for *Physcomitrella patens* (*P. patens*, Pp), *Selaginella moellendorffii* (*S. moellendorffii*, Sm), *Populus trichocarpa* (*P. trichocarpa*, *Pt*), *Sorghum bicolor* (*S. bicolor*, Sb), *Brachypodium distachyon* (*B. distachyon*, Bd), *Arabidopsis thaliana* (*A. thaliana*, At)*,* and *Oryza sativa* (*O. sativa*, Os) were acquired from the downloaded data (ftp://ftp.ensemblgenomes.org/). *MUSCLE* software (http://www.drive5.com) was applied for amino acid (aa) alignments. A phylogenetic tree was generated via the MEGA X software using the neighbor-joining (NJ) method and 1000 bootstrap tests and visualized using the online software tool EvolView [36].

### Homology modeling

The 3D structure of wheat formin proteins was constructed by homology modeling method, which based on the resolved of homologous protein. The Phyre 2 server was been used to obtain the 3D structure and further visualized by PyMOLWin.

### Chromosomal distribution

The location of each *formin* gene on the wheat chromosomes was mapped to IWGSC RefSeq v1.1 (cv. Chinese_Spring) using blast programs (https://blast.ncbi.nlm.nih.gov/Blast.cgi). The centromere locations were retrieved from IWGSC (www.wheatgenome.org). Then, using the MapInspect tool (http://mapinspect.software.informer.com/), their locations were drawn onto the physical map of each chromosome. The graphic of chromosomal distribution was refined using Adobe illustrator.

### Prediction of miRNA target genes

MiRNAs and their targets in *TaFH* genes were predicted via a web-based psRNATarget server using default parameters [37]. They were then visualized using Cytoscape (version 3.7), as described previously [38].

### Identification of the *cis*-acting elements in the *TaFH* genes

The *cis-acting* elements in the 1500 bp upstream promoter regions of the identified *TaFH* genes were analyzed via PlantCARE (http://bioinformatics.psb.ugent. be/webtools/ plantcare/html/). All of these sequences were used to identify the *cis*-acting elements by the recently released *T. aestivum* genome database (http://plant.ensembl.org/, IWGSC RefSeqv1.1).

### Plant materials, treatments, and sample collections

The thermo-sensitive genic male sterile (TGMS) wheat line BS366 was planted in the experimental fields in Beijing (China, N39°54′, E116°18′) and Nanyang (Henan province, China, N39°86′, E116°25′) and managed conventionally. To analyze the roles of *TaFH* genes during pollen development in BS366, we sampled the anthers at three stages: stage 1 (secondary sporogenous cells had formed); stage 2 (all cell layers were present and mitosis had ceased); and stage 3 (meiotic division stage) [27]. For tissue-specific expression analysis, six tissues (root, stem, leaf, glume, stamen, pistil) from BS366 were collected in the field experiment station. Two-week-old wheat seedlings (20 °C, 12-h day/12-h night cycle) were used for abiotic stresses analysis, as per the methods described by Bai et al. [28]. In brief, we sprayed BS366 seedlings with 2 mM SA, 100 mM MeJA, 100 mM GA, 50 mM IAA, and 100 mM ABA. For the high-salinity and drought treatments, the roots of wheat seedlings were soaked in 200 mM NaCl and PEG6000 (− 0.5), and leaf tissues were collected from the seedlings 0, 2, 4, 8, 12, and 24 h post-treatment. To evaluate the low-temperature stress, 2-week-old seedlings were incubated at 10 °C, and leaf tissues were collected from the seedlings at 0, 2, 4, 8, 12, and 24 h time points. All samples were frozen in liquid nitrogen immediately after collection. And a flowchart was provided in Fig. S4.

### Pollen cell and DNA staining

Pollen cell staining was performed as described by Wang et al. [10] and Xu et al. [15]. Briefly, microfilaments and microtubules were respectively marked with tetramethylrhodamine isothiocyanate (TRITC)-phalloidin (Sigma, St. Louis, MO, USA) and anti-α-tubulin (mouse IgG monoclonal anti-α-tubulin, T-9026; Sigma) in pollen cells. DNA staining was conducted using 4′, 6-diamidino-2-phenylindole (DAPI) for counterstaining as described by Xu et al. [15]. After examining the preparations, images were captured using a laser scanning confocal microscope (Nikon A1R, Tokyo, Japan).

### Gene expression assay/analysis

For expression analysis of *TaFH* genes, total RNA was isolated from wheat tissues using TRIzol reagent (Invitrogen, USA) following the manufactures’ instructions. First-strand cDNA synthesis was performed using a PrimeScriptTM RT Reagent kit with gDNA Eraser (TaKaRa, Japan). Next, qRT-RCR assay was conducted on an Eco Real-time PCR system (Illumina, USA) using SYBR® Permix Ex TaqTM (TaKaRa, Japan), as described previously [31].

Relative expression levels of *TaFH* genes were determined using the comparative threshold cycle method 2^-^△△^CT^ [39]. Wheat *18S* gene served as the reference control. All the qRT-PCR reactions were conducted in three biological replicates. The primer used for qRT-PCR were conserved for all three homologues and designed using Primer premier 5.0 program and are listed in Table S2.

## Supplementary Information

**Additional file 1: Figure S1.** Phylogenetic relationship and motif structure of wheat formin proteins. The phylogenetic tree of TaFH proteins constructed from a complete alignment of 25 wheat formin proteins using MEGA 6.0 by the neighbor-joining method with 1000 bootstrap replicates. Domain distribution of TaFH proteins were investigated using the MEME web server.

**Additional file 2: Figure S2.** Exon-intron structures of *TaFH* genes. Exons are represented by green boxes and introns by blank lines.

**Additional file 3: Figure S3.** Three-dimensional structure of representative TaFH protein.

**Additional file 4: Figure S4.** The flowchart of *TaFH* gene family with the treatment of BS366.

**Additional file 5: Table S1.** The interaction of miRNAs with *TaFH* genes. **Table S2.** The primers of the qRT-RCR in TaFHs.

## Data Availability

The data sets supporting the results of this article are included within the article and its additional files. **All the links of datasets** used in the study: The wheat genome Ensemble database (http://plant.ensembl.org/). The Pfam database (http://pfam.sanger.ac.uk/). NCBI–CDD (https://www.ncbi.nlm.nih.gov/cdd/). SMART (http://smart.emblheidelberg.de/). ProtParam (https://web.expasy.org/ ). The formin coding sequences for *P. patens*, *S. moellendorffii*, *P. trichocarpa*, *S. bicolor*, *B. distachyon*, *A.thaliana,* and *O. sativa* were acquired from the downloaded data (ftp://ftp.ensemblgenomes.org). *MUSCLE* software (http://www.drive5.com). IWGSC RefSeq v1.1 (cv. Chinese Spring) (https://blast.ncbi.nlm.nih.gov/Blast.cgi). The location of each formin gene on the wheat chromosomes was mapped to IWGSC RefSeq v1.1 (cv. Chinese_Spring) using blast programs (https://blast.ncbi.nlm.nih.gov/Blast.cgi). The centromere locations were retrieved from IWGSC (www.wheatgenome.org). The MapInspect tool (http://mapinspect.software.informer.com/). PlantCARE (http://bioinformatics.psb.ugent. be/webtools/ plantcare/html/).

## Abbreviations

ABA: Abscisic acid
GA: Gibberellin
IAA: Indole-3-acetic acid
MeJA: Methyl jasmonate
TGMS: Thermo-sensitive genic male sterile
