# Supplementary figures and images for: Comprehensive analysis of formin gene family highlights candidate genes related to pollen cytoskeleton and male fertility in wheat (Triticum aestivum L.)
Source: BMC Genomics. 2021 Jul 24;22:570. doi: 10.1186/s12864-021-07878-7 (PMC8305537; doi:10.1186/s12864-021-07878-7)

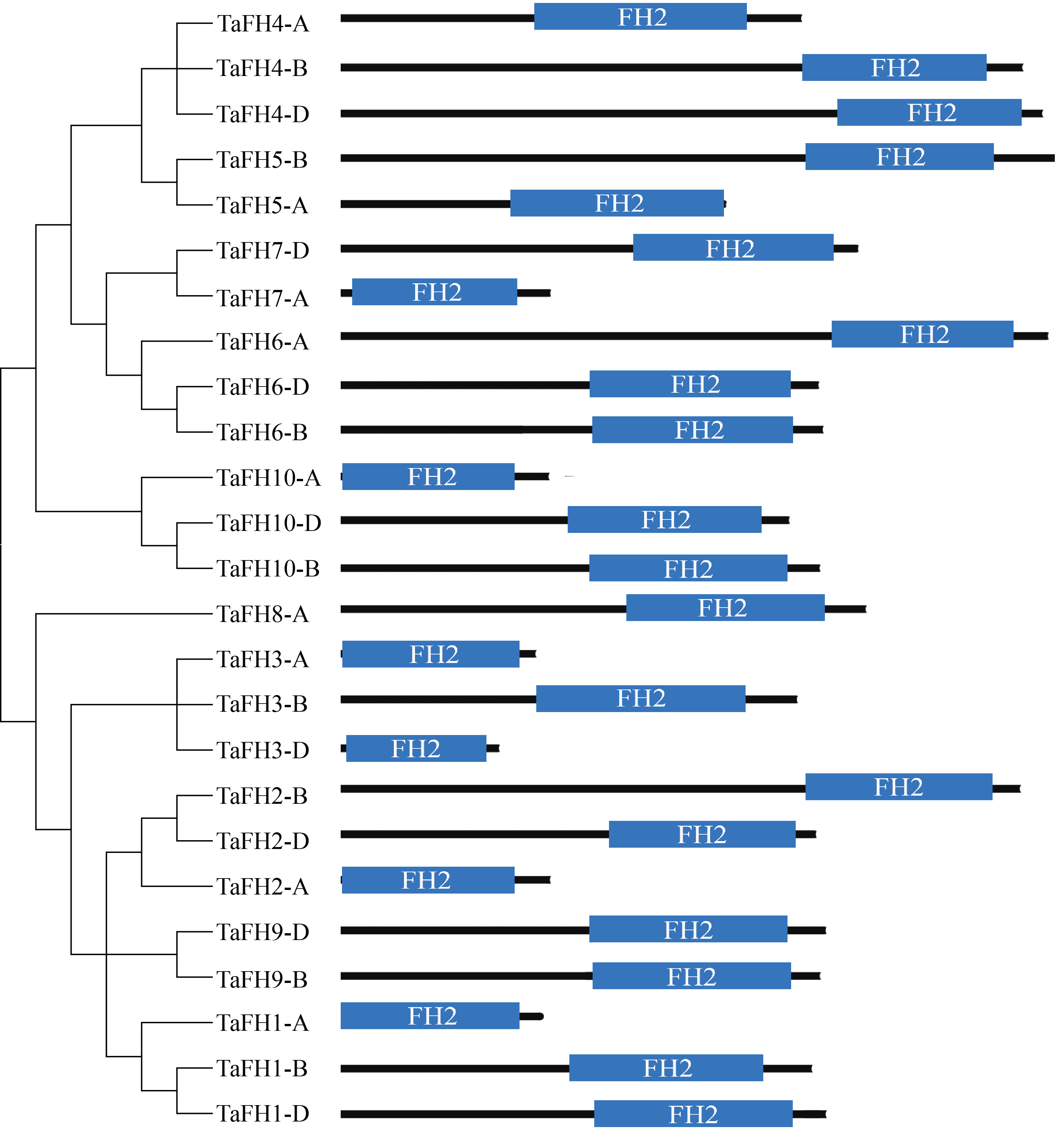

Supplement: Supplementary file 1 — Additional file 1: Figure S1. Phylogenetic relationship and motif structure of wheat formin proteins. The phylogenetic tree of TaFH proteins constructed from a complete alignment of 25 wheat formin proteins using MEGA 6.0 by the neighbor-joining method with 1000 bootstrap replicates. Domain distribution of TaFH proteins were investigated using the MEME web server. [file 12864_2021_7878_MOESM1_ESM.tif]

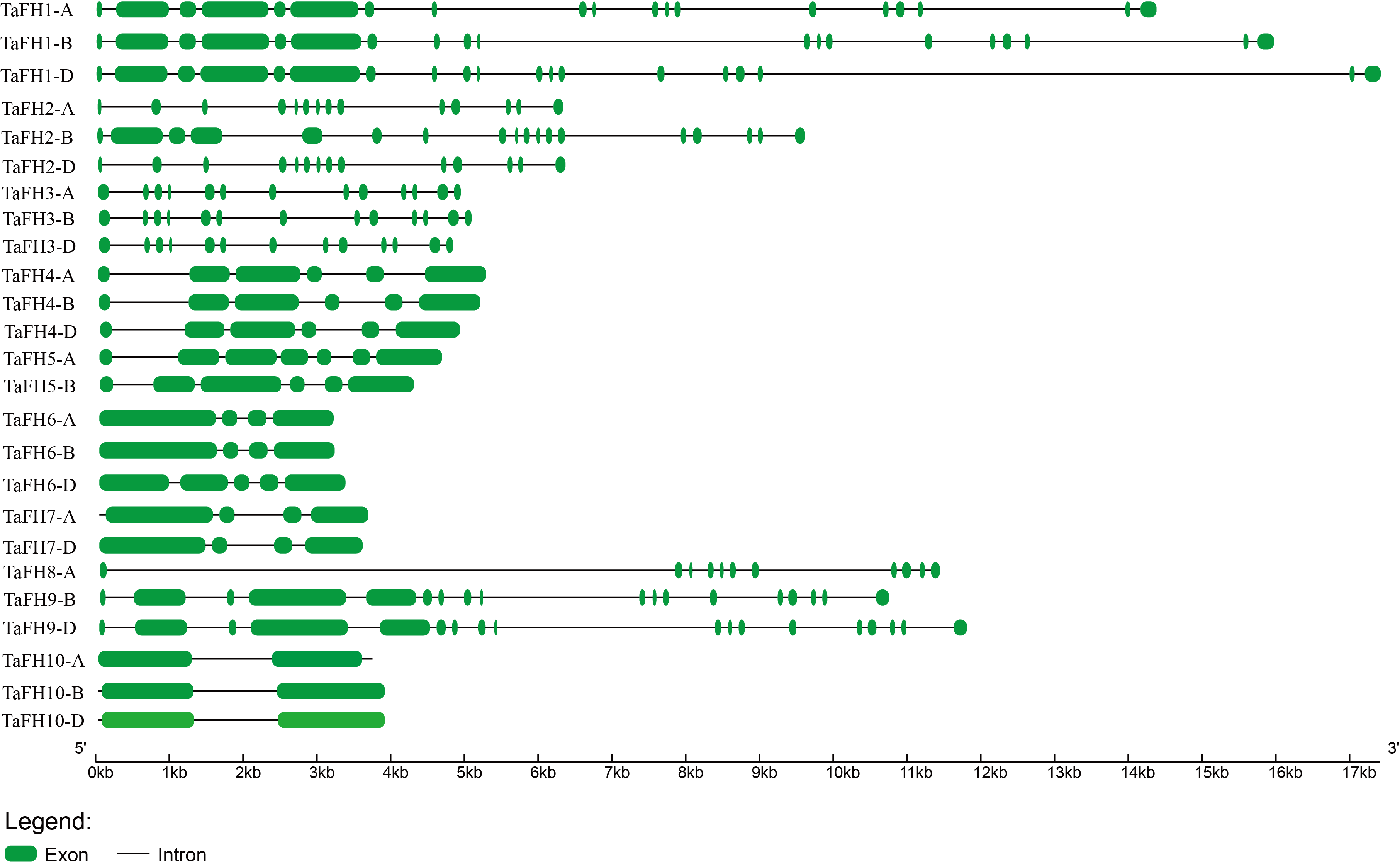

Supplement: Supplementary file 2 — Additional file 2: Figure S2. Exon-intron structures of TaFH genes. Exons are represented by green boxes and introns by blank lines. [file 12864_2021_7878_MOESM2_ESM.tif]

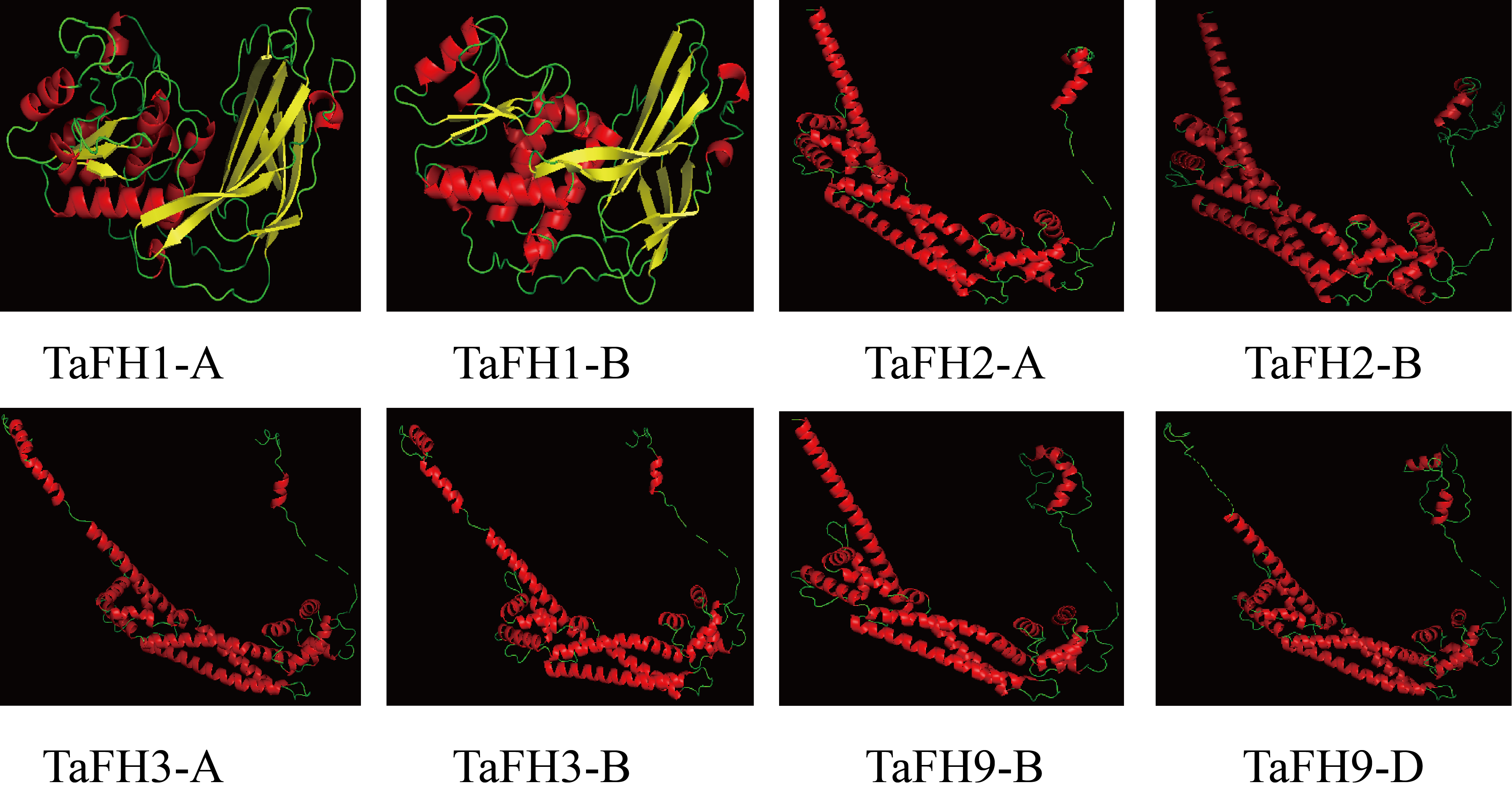

Supplement: Supplementary file 3 — Additional file 3: Figure S3. Three-dimensional structure of representative TaFH protein. [file 12864_2021_7878_MOESM3_ESM.tif]

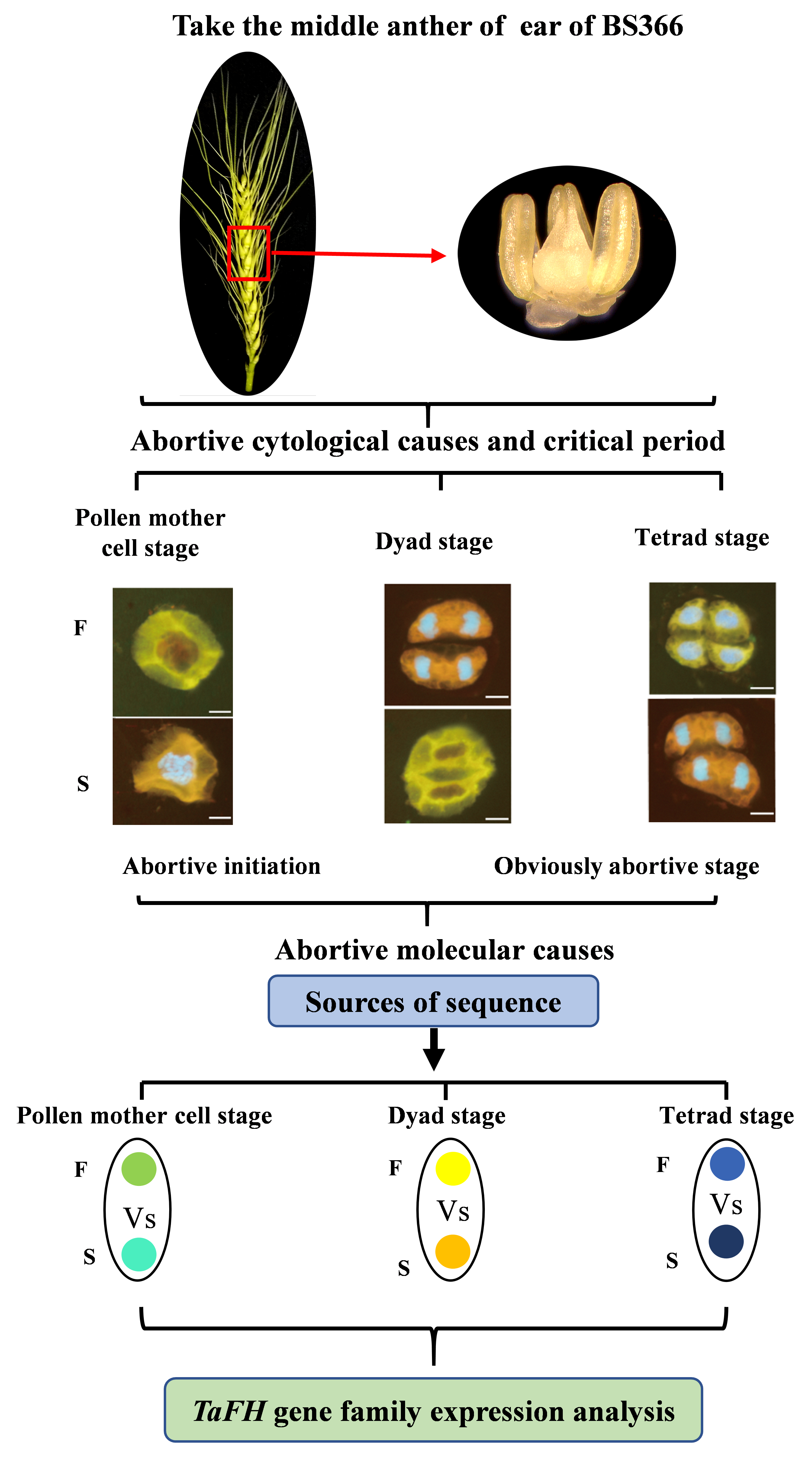

Supplement: Supplementary file 4 — Additional file 4: Figure S4. The flowchart of TaFH gene family with the treatment of BS366. [file 12864_2021_7878_MOESM4_ESM.tif]
